# Clinical Symptoms of Human Rotavirus Infection Observed in Children in Sokoto, Nigeria

**DOI:** 10.1155/2015/890957

**Published:** 2015-11-25

**Authors:** B. R. Alkali, A. I. Daneji, A. A. Magaji, L. S. Bilbis

**Affiliations:** ^1^Faculty of Veterinary Medicine, Usmanu Danfodiyo University, PMB 2346, Sokoto, Sokoto State, Nigeria; ^2^Faculty of Science, Usmanu Danfodiyo University, PMB 2346, Sokoto, Sokoto State, Nigeria

## Abstract

Rotavirus has been identified among the most important causes of infantile diarrhoea, especially in developing countries. The present study was undertaken to determine the occurrence and clinical symptoms of human rotavirus disease among children presenting with varying degree of diarrhoea in selected urban hospitals in Sokoto metropolis, Nigeria. Diarrhoea samples were collected from 200 diarrheic children younger than 5 years of age and tested using a commercially available DAKO Rotavirus ELISA kit which detects the presence of human group A rotaviruses. A questionnaire, based on WHO generic protocol, was completed for each child to generate the primary data. Of the total number of samples collected, 51 were found to be positive for human group A rotavirus indicating 25.5% prevalence of the disease in Sokoto state. The symptoms associated with the disease were analyzed and discussed.

## 1. Introduction

Diarrhoea illnesses were reported to consistently rank as one of the top six causes of all deaths, one of the top three causes of death from infectious disease, and one of the top two causes of death when considering years of life lost [1–3]. Rotavirus was identified to be responsible for up to 20% of these deaths [4]. Also reports have shown that 39% of diarrhoea episodes seen at health centers were rotavirus positive [5, 6].

*Rotavirus* is a genus in the Family of Reoviridae with the characteristic wheel-like (i.e., Rota is Latin for wheel) appearance. The inner capsid contains the viral genome of 11 segments of double stranded RNA that encode six structural and six nonstructural proteins [7]. The structural proteins of the virion are depicted as three concentric circles, forming an equal number of layers around the dsRNA genome (triple layered particle) [8]. It is a nonenveloped triple layered icosahedral virus consisting of an inner core containing proteins VPl, VP2, and VP3, encoded by segments 1–3, a middle capsid made up of protein VP6, encoded by gene segment 6 and an outer capsid made up of a VP7 shell and a VP4 spike protein encoded by segments 7, 8 or 9, and 4, respectively [7]. The external layer of the virus is discontinuous and looks like a sponge, because of the multiple small extensions of the VP4 spike [9].

Rotavirus strains had been classified into eight main (A–H) serotype groups (or serogroups) on the basis of antigenic sites located on the VP6 protein [10]. The most virulent and commonly isolated strains belong to serogroup A (GARVs) as the group constitute an important cause of acute infectious diarrhoea in children and various domestic mammalian and avian species.

Indeed group A rotaviruses were reported to constitute the major cause of severe gastroenteritis in young children and animals worldwide affecting nearly all animals from whales and snakes to cows and pigs [11, 12]. Studies have also shown that by the age of two years almost all children are infected by rotavirus with children in industrialized countries experiencing their first infection at comparatively older age compared to those in developing countries [5, 13].

In Nigeria, a high incidence of childhood diarrhoea is estimated to account for over 160 000 of all deaths in children less than 5 years of age annually and of this number approximately 20% had been associated with rotavirus infection [14]. Although diarrhoea, vomiting, and dehydration are frequently associated with the disease, there is need to comprehensively evaluate the symptoms and signs associated with rotavirus disease especially because various pathogens have been identified to cause severe diarrhoeal diseases including viruses and bacteria. Thus, the study was designed to provide baseline information and insight into the general symptoms of rotavirus disease and identify the symptoms that may be significantly associated with the disease among children in Sokoto, Nigeria.

## 2. Study Area

The study was conducted in three urban hospitals located in Sokoto state, namely, Usmanu Danfodiyo University Teaching Hospital, Sokoto (UDUTH), Specialist Hospital, Sokoto, and Women and Children Hospital, Sokoto. These urban hospitals also service rural communities from all parts of the state, including neighboring states. Sokoto state lies between longitude 11° 30′ to 13° 50′ East and latitude 4° to 6′ North. The state falls within the savannah zone and is located in northwestern Nigeria where life expectancy for men and women is 51 years and 52 years, respectively. The GNP per capita is 320 dollars.

### 2.1. Sampling Method

Simple random sampling method was adopted in the study where each child in the population had equal chance of being selected. This sampling technique provided opportunity in the realistic generalization of the research population. A questionnaire based on WHO generic protocol was administered to generate the primary data along with sample bottle where adequate information on every child was obtained. Patient information such as identification number, address, and admission diagnosis, date of admission, and presenting symptoms were collected. In order to enhance the validity of the research questionnaire, the instrument was validated by both validity and reliability tests. The validity of the questionnaire was determined by the critique of the research experts of the questionnaire. The modification of the questionnaire was based on the experts' comments and advice. The reliability of the questionnaire was determined through the administration of the modified copy to some nurses and matrons of the hospitals selected for the study. The results provided the basis for the final modification of the questionnaire.

## 3. Data Analysis

### 3.1. Samples Collection

Statistical Programme for Social Sciences (SPSS17.0) was used to analyze the data. Data was analyzed by simple inferential statistics. The frequencies of findings and the percentages they represent were highlighted on tables, graphs, and charts. Also Chi-square analysis was used for significance testing in drawing inferences.

Diarrhoea samples were collected from all diarrheic children under 5 years of age that were presented at the identified hospitals after obtaining parental consent. Diarrhoea in the study was defined as the passage of more than 3 looser than normal stools within 24 hours. The stool samples were collected aseptically in sterile commercial bijou bottles, adequately labeled (patient ID and date of collection), and transported on ice to the Veterinary Microbiology Laboratory of Usmanu Danfodiyo University, Sokoto, where they were stored at −20°C until they were transported on ice to Noguchi Memorial Institute for Medical Research (NMIMR) in Accra, Ghana, where they were stored at −20°C until they were tested. A stool specimen logbook was kept in the laboratory where information on all diarrhoeal children was checked regularly and matched with the information in the questionnaire to ensure proper entry of information. Also, data form for analysis of rotavirus diarrhoea was adapted from the WHO generic protocol with some modifications.

## 4. Determination of Rotavirus Antigen by ELISA

A commercial DAKO Rotavirus ELISA kit was used to detect the presence of human group A rotaviruses in stool samples according to the manufacturer's instructions. Briefly, 2 drops (100 *μ*L) of each of the prepared 10% stool suspension were added into each well of the provided 96-well microtiter plate precoated with rotavirus specific rabbit polyclonal antibody except the first three wells designated as blank, negative, and positive controls, respectively. Two drops of the conjugate contained in the kit were then added into each microwell and mixed gently by swirling on table's top. The plates were then incubated at room temperature for 1 hour. The contents were then discarded and the plates were tapped upside down against paper towel to remove all liquid from the wells. The wells were then overflowed with freshly prepared washing buffer and contents were discarded. The plates were tapped upside down against paper towel to remove excess wash buffer. The washing was repeated 5 times. Two drops of the substrate contained in the kit were then added to each microwell and the plate was incubated at room temperature for 10 minutes. Results were then observed visually within 10–20 minutes after the incubation. Finally the reaction was stopped by the addition of stopping solution (H_2_SO_4_) to each microwell and the results were finally read spectrophotometrically within 30 minutes of stopping the reaction on Multiskan ELISA reader (Multiskan Plus, Labsystems Oy, Pulttitie 8, P.O. Box 8, 00881 Helsinki, Finland) at a wavelength of 450 nm.

## 5. Interpretation of the Results

### 5.1. Visual Observation

All negative controls were colourless or faintly blue while samples with a more intense blue colour than negative control were observed as positive. Samples that showed equal or less colour than the negative control were observed as negative.

### 5.2. Photometric Determination/Readings

The negative control or mean of the negative controls should be less than 0.15 absorbance units. The cutoff value was calculated by adding 0.100 absorbance units to the negative control value. All samples with absorbance value above the cutoff value were read as positive while all samples with absorbance value below the cutoff point were read as negative.

## 6. Results

### 6.1. Rate of Rotavirus Detection among Children in Sokoto, Nigeria

Out of the 200 human diarrhoea stools examined by ELISA, rotavirus was detected in 51 of the samples, indicating a prevalence of 25.5%.

### 6.2. Stool Analysis of Rotavirus Diarrhoea in Children in Sokoto

**Figure 1:**
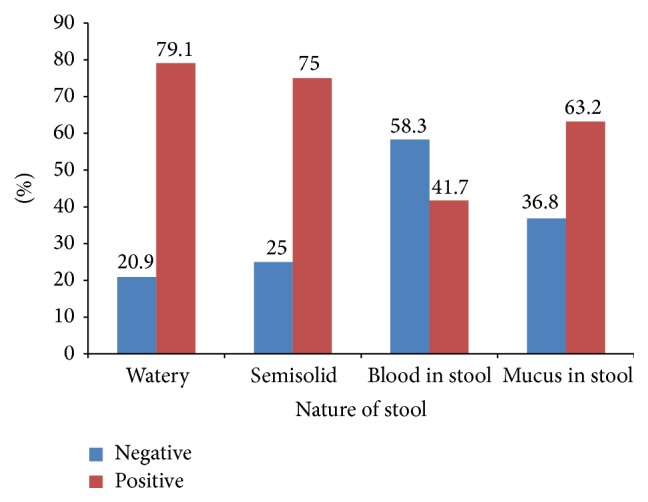
Distribution of rotavirus diarrhoea in children presenting with different types of stool in Sokoto.

Figure 1 showed the summary of data on the frequency of rotavirus detection according to the nature of stools. The data showed a high frequency of detection in watery stool tinged with blood (58.3%) indicating possible mixed infection with other parasites. The detection of the virus in stool mixed with mucus was 36.8% which further supports the possibility of mixed infection.

### 6.3. Analysis of Duration of Rotavirus Diarrhoea in Children in Sokoto

The results showed that, for the 51 rotavirus positive children, diarrhoea lasted for 2 days in majority of cases (43.1%). However, the diarrhoea could last for up to 7 days as observed in 27.5% of rotavirus positive children. Only in few cases (2%) did the duration of the diarrhoea reach 10 days (Table 1).

### 6.4. Analysis of Vomiting in Rotavirus Diarrhoea in Children in Sokoto

The results showed that vomiting was present in over 78.4% of all rotavirus diarrhoea while vomiting was absent in 22.6% of the cases (Table 2). Chi-square analysis indicated significant association between rotavirus diarrhoea and vomiting (*P* < 0.05). The duration of vomiting in days observed in 51 rotavirus positive children showed that majority of cases occurred within 1-2 days (90%) with very few cases occurring up to seven days (7.5%) (Table 3).

### 6.5. Analysis of Dehydration in Rotavirus Diarrhoea in Children in Sokoto

**Figure 2:**
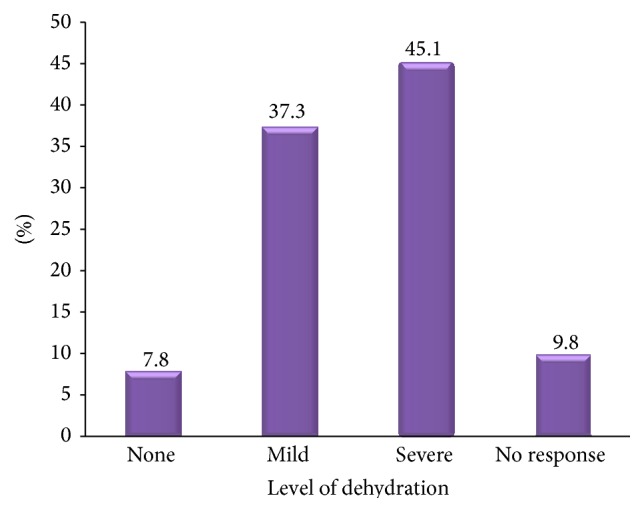
Dehydration status of rotavirus diarrhoea positive children in Sokoto.

The data on the level of dehydration in rotavirus diarrhoea positive children in Sokoto showed that none, mild, or severe dehydration was present in 7.8%, 37.3%, and 45.1%, respectively, as summarized in Figure 2. The result showed that the level of dehydration in the majority of children suffering from rotavirus diarrhoea was severe. Chi-square analysis also indicated statistically significant association between rotavirus diarrhoea and dehydration (*P* < 0.05).

### 6.6. Analysis of Other Symptoms Present in Rotavirus Diarrhoea in Children in Sokoto 

The data indicated that majority of the children suffering from rotavirus diarrhoea had either fever (72.5%) or fever and respiratory symptoms (11.8%). The prevalence of rotavirus diarrhoea in children showing respiratory symptoms without fever was 3.9% (Table 4). Chi-square analysis did not indicate any significant association between rotavirus diarrhoea and these symptoms (*P* > 0.05).

## 7. Discussion

World Health Organization (WHO) estimated that 42 percent of the total 10.6 million deaths among children younger than five years of age worldwide occur in the African region [15]. Although mortality rates among these children had declined globally, the situation in Africa was considered strikingly different [16]. This was because the mortality rate of children younger than 5 years of age in the African region was said to be seven times higher than that in the European region [16]. Furthermore, earlier report by Cunliffe et al. [5] showed that, of the 25 million children born each year in sub-Saharan Africa, 4.3 million (about 1 in 6) would die by the age of 5 years and about 1/5 of these deaths (850,000) would be from diarrhoea. Interestingly, rotavirus was identified to be the single most important pathogen associated with diarrhoea cases in both hospital patients and outpatients [5].

In this study, 51 (25.5%) out of the 200 diarrhoeic children tested were found to be positive for rotavirus while 149/200 (74.5%) tested negative for rotavirus. Thus, the prevalence of rotavirus diarrhoea accounted for 25.5% of diarrhoea cases among children younger than five years of age presented to hospitals in Sokoto metropolis.

The result of this study is consistent with the sentinel based rotavirus surveillance system and hospital based study results within the African region [17].

Interestingly, however, earlier studies carried out in different parts of northern Nigeria reported low prevalence. Pennap and Umoh [18] reported rotavirus infection prevalence of 15.6% among children (0–60 months old) that presented with diarrhoea in northeastern Nigeria. Aminu et al. [19] similarly reported rotavirus prevalence of 18% among diarrheic children and 7.2% among nondiarrheic children in a hospital setting in northern Nigeria and prevalence of 9% among children younger than five years of age in a community based study in the same region. Similarly, other investigators reported lower prevalence of the infection in the northern region [20]. The low prevalence reported in the community based study is expected as higher prevalence of rotavirus infection is more likely to be encountered in hospital based studies since rotavirus positive cases are often severe and likely represented in hospitals [21]. However, generally, studies from southern Nigeria had shown higher rotavirus prevalence values than those from northern Nigeria [22–25]. The differences in the prevalence recorded by different investigators had been attributed to differences in time of sample collection, method of screening samples, geographical location of the study, or changing trends of the burden of the rotavirus disease over the years [26].

Earlier studies indicated that stools in rotavirus diarrhoea were nonbloody and generally lack faecal leukocytes and mucus may be found in about 20% of cases [27, 28]. But surprisingly the result in this study showed a high frequency of rotavirus detection in watery stool tinged with blood (58.3%). This is also in contrast with the recent observation that blood tinged diarrhoea was rare in rotavirus infection [18]. However, the observation of high prevalence of rotavirus in blood watery stool may likely be a result of mixed infection with other pathogens such as* Shigella* because, in developing areas like Sokoto, transmission of enteric pathogens and coinfection are high as a result of poor sanitation, low immunity, lack of access to treatment, imbalanced diet, and poor nutrition. The detection rate of the virus in stool mixed with mucus in this study was 36.8% which further supports the possibility of mixed infection even though stool in rotavirus infection had been reported to often contain large amounts of mucus [29].

The result on the occurrence of vomiting in children with rotavirus diarrhoea showed that vomiting was present in over 33% of all rotavirus positive children while vomiting was absent in 13.8% of the cases. There was significant association between vomiting and rotavirus diarrhoea (*P* < 0.05). Indeed, vomiting had always been a common occurrence in rotavirus diarrhoea and had been reported to precede the diarrhoea in approximately half of all rotavirus diarrhoea cases [30]. The duration of vomiting in days observed in the rotavirus positive children showed that majority of cases occurred within 1-2 days (90%) with very few cases occurring up to seven days (7.5%). This is in agreement with the observation of Pennap and Umoh [18]. But, generally rotavirus disease is usually self-limiting, lasting for four to eight days, and the overall duration of symptoms was reported to be between 2 and 22 days [31]. Recent report showed that, in severe rotavirus cases, children may suffer from symptoms of gastroenteritis for up to 9 days and then recover [32].

Rotavirus had often been associated with severe dehydration which is actually responsible for death associated with the infection [33]. In addition, children with dehydration had been found to be about two times more likely to have rotavirus diarrhoea [6]. In this study, the prevalence of rotavirus diarrhoea in children with none, mild, or severe dehydration was found to be 15.9%, 17.8%, and 42.4%, respectively. The result showed that the level of dehydration in the majority of children suffering from rotavirus diarrhoea was severe. Chi-square analysis also indicated significant association between rotavirus diarrhoea and dehydration (*P* < 0.05). The result is in conformity with the report of Pennap and Umoh [18]. Indeed, rotavirus infection had been associated with severe diarrhoea episodes and vomiting which often led to severe dehydration in babies and young children [33].

The analysis of other symptoms observed with rotavirus diarrhoea in children in Sokoto showed that the majority of the children suffering from rotavirus diarrhoea had either fever (26.8%) or fever and respiratory symptoms (25%). The prevalence of rotavirus diarrhoea in children showing respiratory symptoms without fever was 21.1%. Chi-square analysis did not indicate any significant association between rotavirus diarrhoea and these symptoms (*P* > 0.05). When the frequency of occurrence of fever was considered alone or in combination with respiratory symptoms, the result showed that fever was present in 51.8% of the cases. This is in consonance with many reports that indicated presence of fever in about 45%–84% of patients suffering from rotavirus diarrhoea [34–37]. The observation of the presence of respiratory symptoms in 25% of the cases is also in agreement with earlier reports that indicated presence of various upper and lower respiratory infections, including otitis media, laryngitis, pharyngitis, and pneumonia during rotavirus illness [38–40].

## 8. Conclusion

Rotavirus detection was the greatest in children with blood tinged watery stool indicating high possibility of mixed infections occurring in this environment. The symptoms of vomiting and dehydration were significantly associated with rotavirus diarrhoea while other symptoms such as fever and/or respiratory symptoms singly or in combination occur in rotavirus diarrhoea but are not significantly associated with the disease.

## Figures and Tables

**Table 1:** Duration of rotavirus diarrhoea in children in Sokoto.

Duration of diarrhoea in days	Number of positive cases	% positive	Cumulative %
0–2	22	43.1	43.1
3-4	12	23.5	66.7
5–7	14	27.5	94.1
8–10	2	3.9	98
>10 days	1	2	100

Total	51	100	

**Table 2:** Frequency of vomiting in rotavirus diarrhoea in children in Sokoto.

Vomiting	Number of positive cases	Percentage positive
Yes	40	78.4
No	11	21.6

Total	51	100.0

**Table 3:** Duration of vomiting in rotavirus diarrhoea in children in Sokoto.

Duration of vomiting in days	Number of positive cases	% positive	Cumulative %
No response	11	0	0
0–2	36	90	90
3-4	1	2.5	92.5
5–7	3	7.5	100

Total	51	100	

**Table 4:** Presence of other symptoms in rotavirus diarrhoea in children in Sokoto.

Other symptoms present	Frequency	Percent	Cumulative percent
Fever	37	72.5	82.2
Respiratory symptoms	2	3.9	86.7
Respiratory symptoms and fever	6	11.8	100.0
Total	45	88.2	
No response	6	11.8	

Total	51	100.0	
